# MRI-Based Radiomic Features Help Identify Lesions and Predict Histopathological Grade of Hepatocellular Carcinoma

**DOI:** 10.3390/diagnostics12051085

**Published:** 2022-04-26

**Authors:** Valentina Brancato, Nunzia Garbino, Marco Salvatore, Carlo Cavaliere

**Affiliations:** IRCCS Synlab SDN, 80143 Naples, Italy; valentina.brancato@synlab.it (V.B.); direzionescientifica.irccssdn@synlab.it (M.S.); carlo.cavaliere@synlab.it (C.C.)

**Keywords:** hepatocellular carcinoma, radiomics, MRI

## Abstract

Hepatocellular carcinoma (HCC) is the most common form of liver cancer. Radiomics is a promising tool that may increase the value of magnetic resonance imaging (MRI) in the management of HCC. The purpose of our study is to develop an MRI-based radiomics approach to preoperatively detect HCC and predict its histological grade. Thirty-eight HCC patients at staging who underwent axial T2-weighted and dynamic contrast-enhanced MRI (DCE-MRI) were considered. Three-dimensional volumes of interest (VOIs) were manually placed on HCC lesions and normal hepatic tissue (HT) on arterial phase post-contrast images. Radiomic features from T2 images and arterial, portal and tardive post-contrast images from DCE-MRI were extracted by using Pyradiomics. Feature selection was performed using correlation filter, Wilcoxon-rank sum test and mutual information. Predictive models were constructed for HCC differentiation with respect to HT and HCC histopathologic grading used at each step an imbalance-adjusted bootstrap resampling (IABR) on 1000 samples. Promising results were obtained from radiomic prediction models, with best AUCs ranging from 71% to 96%. Radiomics MRI based on T2 and DCE-MRI revealed promising results concerning both HCC detection and grading. It may be a suitable tool for personalized treatment of HCC patients and could also be used to develop new prognostic biomarkers useful for HCC assessment without the need for invasive procedures.

## 1. Introduction

Hepatocellular carcinoma (HCC) is the most common form of liver neoplasia and is one of the most common causes of tumor deaths worldwide, accounting for 75–85% of primary liver cancers [1]. Early diagnosis and accurate staging assessment are crucial in the management of HCC, primarily to optimize the treatment and improve prognosis [2,3]. According to the degree of differentiation of cancer cells, HCC can be classified into poorly differentiated HCC, moderately differentiated HCC and well-differentiated HCC [4,5]. Previous studies have reported that the overall survival rate of patients with HCC from well- to moderately differentiated HCC was higher than that of patients with poorly differentiated HCC, and the risk of recurrence was lower [4,6]. HCC grade is usually confirmed by postoperative pathologic examination of tumor samples [7]. However, the preoperative evaluation of the HCC differentiation degree is of critical importance in view of personalized treatment options [4]. Liver biopsy is now the most common procedure to obtain information on HCC grade in the preoperative setting [8]. However, it is an invasive procedure and is susceptible to sampling errors [9,10]. Imaging plays a crucial role in HCC diagnosis and grading [2]. Currently, all major clinical practice guidelines recommend the use of multiphasic computed tomography (CT) and magnetic resonance imaging (MRI) with extracellular contrast agents as the first-line imaging modalities for HCC diagnosis and staging [11,12,13]. Although CT is largely available, rapid, and requires less expertise in performing and interpreting images than MRI, its drawbacks include radiation exposure and the relatively low contrast associated to soft tissues, that obliges to use iodinate contrast agents. Conversely, the higher soft-tissue contrast of MRI allows the evaluation of a large number of tissue properties that may be useful for HCC management [12,13,14]. In dynamic contrast-enhanced MRI (DCE-MRI), the signal intensity during the arterial, portal venous, and delayed phases reflect differences in the distribution of contrast agent between the vascular and extravascular spaces in liver tumors and parenchyma [15]. This technique is gaining importance for diagnosis and staging of HCC and is considered as the most sensitive method for detecting small HCC lesions and precancerous nodules that are considered to have a high risk for developing HCC [12,13,16]. Information from DCE-MRI can be supplemented with other MRI sequences, which can help to comprehensively assess the liver of patients at risk of HCC [17,18]. Recent studies have shown that the addition of T2-weighted imaging to DCE-MRI can improve the diagnostic performance of MRI in the detection of HCC compared to DCE-MRI alone. This could be particularly useful for small lesions (<1–2 cm) since they may show hypervascularity but not washout, thus increasing the suspicion for underlying HCC [17,19,20]. Thus far, the imaging evaluation of HCC has been mostly based on the assessment of tumor size and the subjective interpretation of qualitative descriptors, which are prone to variations [19,20]. Chang et al. [21] found that low arterial enhancement on DCE-MRI and low ADC were associated with worse histological HCC grades. An et al. [22] found that a qualitative approach based on DWI and subtracted DCE-MRI helped predict HCC grades. These studies suggest that the different pathological features between HCC and other liver lesions, as well as those between different HCC grades, could be reflected in MRI. However, pathological features are difficult to distinguish with the naked eyes. Moreover, this process is operator dependent, subjective, time-consuming, and lacking in reproducibility. In the last years, to improve image-based HCC detection and characterization, the use of quantitative image descriptors is gaining more and more popularity in the research field. This approach is called radiomics and consists of the extraction of a large number of features from the imaging data, which are supposed to contain information reflecting the underlying tissue biology [23,24,25]. In the past decade, radiomics studies for management of HCC patients have substantially increased, with most of them aiming at assessing the power of radiomic features for prediction of microvascular invasion, overall survival [26], recurrence and treatment response [27,28,29]. Recent studies aimed at reviewing the state of the art in radiomics of HCC, highlighting the main principles, clinical applications, and limitations [30,31,32,33,34]. However, what emerges from these works is that the majority of radiomic studies on HCC were based on CT, and only a few of them investigated multiparametric MRI [35,36]. In addition, there are few studies evaluating the power of MRI radiomic features in discriminating the differentiation degree of HCC [37,38,39,40]. However, linking robust radiomics features with histopathological findings could improve clinical decision making without resorting to invasive procedures. Therefore, using the publicly available LIHC cohort from The Cancer Imaging Archive (TCIA) [41,42,43], we aimed at investigating the ability of radiomic features extrapolated from preoperative T2 and DCE-MRI in both differentiating normal liver tissue from HCC and predicting HCC histological grade.

## 2. Materials and Methods

### 2.1. Study Population

A total of 237 studies of 97 patients with histopathologically proven HCC and the associated clinical data were downloaded from The Cancer Imaging Archive Liver Hepatocellular Carcinoma (TCGA/TCIA-LIHC) data collection [41,42,43]. Among these, 38 patients were selected according to the following criteria: acquisitions including both dynamic MRI with contrast medium injection and T2, patients that had not received prior treatment for their disease (ablation, chemotherapy, or radiotherapy). Exclusion criteria included: patients with artifacts on MR images, incomplete imaging data, incomplete clinical data that are functional to the study aim. Characteristics of included patients are reported in Table 1.

### 2.2. MRI Acquisition Protocol

All MRI examinations were performed using 1.5 T MRI machine (19 studies on Siemens equipment (Munich, Germany) and 18 on GE Medical Systems device (Wauwatosa, WI, USA)) with a dedicated phased-array body coil. A standard abdominal MRI protocol containing following sequences was acquired: axial fat-suppressed T2-weighted (repetition time (TR) = 4500 ms, echo time (TE) = 751 ms, slice thickness = 4 mm, matrix size = 384 × 384), a spoiled GRE 3D sequence for DCE-MRI (TR = 4.48 ms, TE = 1.632 ms, slice thickness = 3 mm, matrix size = 512 × 512).

### 2.3. Image Preprocessing and 3D ROIs Segmentations

Three-dimensional volumes of interest (VOIs) encompassing the HCC were manually obtained by using ITK-SNAP (version 3.6.0, http://www.itksnap.org, accessed on 29 October 2021) on the arterial phase by a radiologist with 8 years of experience. The round brush shape tool was used to segment the HCC on the axial plane, slice by slice, with the possibility to visualize the extension of lesion on the coronal and sagittal planes. VOIs were also drawn in the healthy liver tissue (HT) on the arterial phase images of the same patients. In this case, VOIs were drawn on three consecutive slices of a liver small portion, being careful to exclude the blood vessels. T2 images were all resliced on arterial phase images. During the segmentation procedure, the radiologist was blinded to all clinical information relative to the included patients. Prior to radiomic features extraction, normalization was applied on both three phases from DCE-MRI images and T2 images intensities. Specifically, the intensities were normalized by centering them at their respective mean value with standard deviation of all gray values in the original image [50,51].

### 2.4. Radiomic Analysis

#### 2.4.1. Radiomic Features Extraction

**Figure 1:**
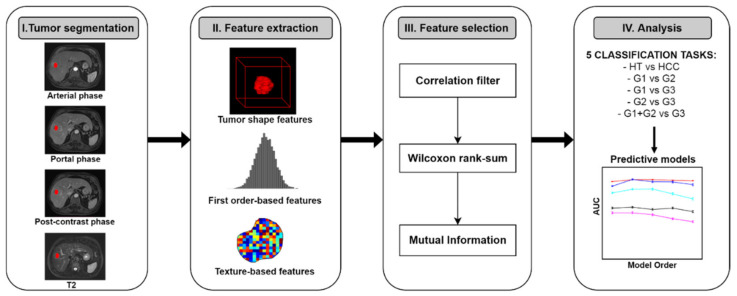
The workflow of radiomics analysis used in this study. Abbreviations: G1, well-differentiated HCC; G2, moderately differentiated HCC; G3, poorly differentiated HCC; AUC, area under the receiver operating characteristic curve.

The radiomic workflow of the study is summarized in Figure 1. A total of 386 radiomics features were extracted from segmented VOIs (both HCC and HT) by using the open source Python package Pyradiomics [52] (https://pyradiomics.readthedocs.io/en/latest/, accessed on 16 December 2021). The extracted radiomics features were categorized into three groups: shape features (n = 14), first-order features (n = 18) and texture features (n = 75).

First order and textural features were extracted from each MRI investigated sequence (T2 and arterial, portal, and tardive post-contrast acquisition). A detailed list of the extracted radiomics features are listed in the Supplementary Materials (Table S1). The computing algorithms can be found at www.radiomics.io (accessed on 16 December 2021), and the image biomarker standardization initiative (IBSI) presents a document to standardize the nomenclature and definition of radiomic features [53]. Refer to Supplementary Materials (Section S2) for the Pyradiomics parameter file used for feature extraction.

**Figure 2:**
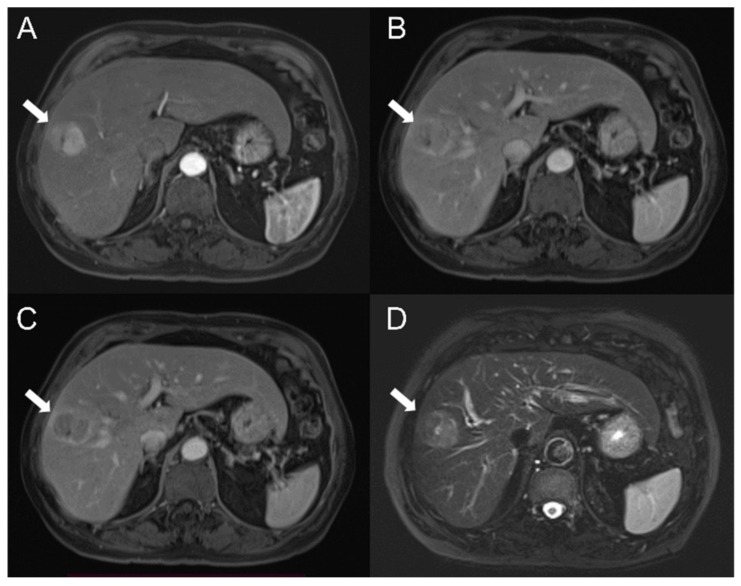
Well-differentiated hepatocellular carcinoma (HCC) (G1) in the right hepatic lobe of a 65-year-old white male. Results showed a hyperintense lesion on axial arterial phase (**A**) and the same lesion appeared hypointense on axial portal and tardive phases (**B**,**C**) and on fat-suppressed axial T2-weighted sequence (**D**) (white arrow).

**Figure 3:**
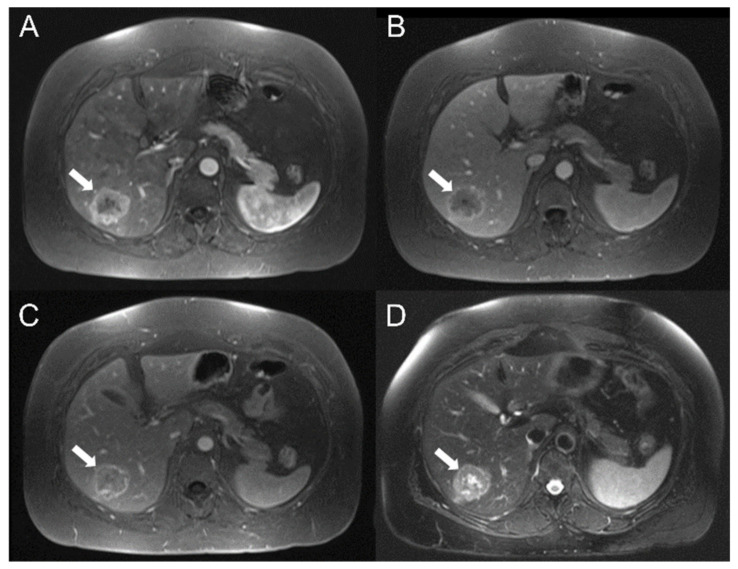
Moderately differentiated HCC (G2) in the right hepatic lobe of a 54-year-old white female. Results showed hypointense mass on axial dynamic study (**A**–**C**), and hyperintensity on the lesion central part on fat-suppressed axial T2-weighted sequence (**D**) (white arrow).

**Figure 4:**
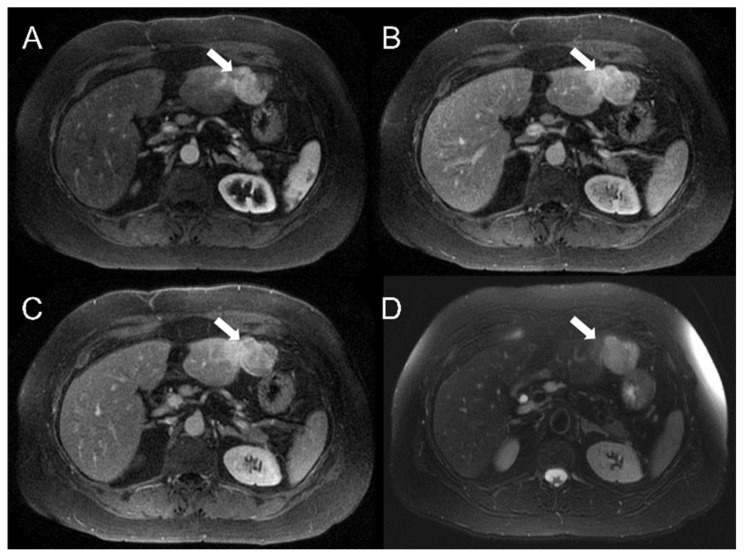
Poorly differentiated HCC (G3) in the left hepatic lobe of a 57-year-old white female. Results showed hyperintense HCC on axial dynamic acquisition (**A**–**C**) and on fat-suppressed axial T2-weighted sequence (**D**) (white arrow).

Five classification tasks were investigated: HCC vs. HT, G1 + G2 vs. G3, G1 vs. G2, G1 vs. G3, G2 vs. G3, with G1, G2 and G3 standing for well-, moderately and poorly differentiated HCC, respectively. Supplementary analyses were also performed to investigate the ability of T2 and DCE-MRI radiomic features in predicting AJCC stage (See Supplementary Section S6). Procedures described in the following two paragraphs are to be considered per classification task. Examples of well-differentiated, moderately differentiated and poorly differentiated HCC are shown in Figure 2, Figure 3 and Figure 4, respectively.

#### 2.4.2. Radiomic Feature Selection

Feature selection was performed in three steps. In the first step, a correlation filter based on the absolute values of pairwise Spearman’s correlation (ρ) coefficient was used to reduce feature redundancy. Threshold for ρ was set to 0.8. Briefly, if two features had ρ > 0.8, the variable with the largest mean absolute correlation is removed. The second step involved a further feature restriction through a univariate analysis and was performed by using non-parametric Wilcoxon rank-sum test to investigate the statistical significance with respect to the outcome. For the HCC/HT classification task, step II was also performed by means of the paired Wilcoxon signed-rank test to explore the same task in a paired setting. The significantly different features were then selected. The third step consisted of ranking the remaining features based on the mutual information (MI) between the distribution of the values of a certain feature and the membership to a particular class. Features are evaluated independently, and the final feature selection occurs by aggregating the 5 top ranked ones [54,55,56]. All steps were implemented using Matlab R2020a (The MathWorks Inc., Natick, MA, USA).

#### 2.4.3. Multivariable Prediction Models Building and Analysis

For each classification task, the reduced feature set was used to build logistic regression models of order *m* from 1 to 5 that would best predict the presence of HCC and its grade using an imbalanced-adjusted bootstrap resampling (IABR) approach on 1000 bootstrap samples [57] that, one by one, added to the *m*th model the feature that maximized the 0.632 + bootstrap area under the receiver operating characteristic curve (AUC) of the models of order *m*. Specifically, 1000 bootstrap samples were randomly drawn with replacement from the available dataset and used as training set. The testing set consisted of the instances not appearing in the bootstrap sample. Then, the probability of picking a positive and a negative instance in the bootstrap sample was made the same by applying the imbalance-adjustment step [58,59].

For each model order, the combination of features maximizing the 0.632+ area under the receiver operating characteristic curve (AUC) within 1000 bootstrap training and testing samples was identified. Finally, IABR on 1000 samples was performed again for all models in order to evaluate prediction performances [57,60].

Finally, for each classification task, the prediction model was obtained choosing the order that maximize the AUC and computing the final model logistic regression coefficients for the selected combination of features according to the following equation [57]:

(1) $$g\left(x_{i}\right)\;{=\beta }_{0}+\overset{v}{\underset{j=1}{\sum }}\beta_{j}x_{ij},\;\mathrm{for}\;i=1,\;2,\;\ldots ,\;N,$$

Equation (1) represents a multivariable model composed by a linear combination of p variables where x_i_ is the vector of input variables (radiomic features) of the *i*th patient, N is the total number of patients, and β is the set of regression coefficients of the model that are calculated by means of a logistic regression model described by the following equation [57]:

(2) $$g\left(x_{i}\right)=P\left(y_{i}{=1|x}_{i}\right)=\frac{\exp[g\left(x_{i}\right)]}{1+\exp[g\left(x_{i}\right)]},\;\mathrm{for}\;i=1,\;2,\;\ldots ,\;N,$$

such that the conditional probability of the set of binary outcome values {0,1} given the input data x_i_ is maximized for i = 1.

DeLong method with Bonferroni correction was applied to compare the predictive ability of the resulting logistic regression models [61].

## 3. Results

### 3.1. Radiomic Features Selection

Considering the HCC/HT classification task, step I of feature selection returned 48 radiomic features. Then, Wilcoxon rank-sum test used in step II of feature selection (unpaired setting) revealed significant results for 29 radiomic features, of which there were two shape features, eight features extracted from arterial images, four from portal images, ten from T2 images and the remaining five from tardive images (see Supplementary Table S2). In a paired setting, paired Wilcoxon signed-rank test revealed significant results for the same 29 features and five additional features: three extracted from T2 images, one from arterial images and 1 from portal images (see Supplementary Table S3). The top five features selected after the MI-based feature selection step (step III) are listed in Table 2 and were the same both considering paired and unpaired setting. For the G1 + G2/G3 classification task, the step I of feature selection returned 52 radiomic features. Then, Wilcoxon rank-sum test used in step II of feature selection revealed significant results for six radiomic features, of which there was one from arterial images, one from portal images, one from tardive images and three from portal images (see Supplementary Table S4). The top five features selected after the MI-based feature selection step (step III) are listed in Table 2. Finally, concerning the classification tasks aiming at predicting differences between G1, G2, and G3 among each other, step I and step II of feature selection returned, respectively, 49 and 6 (for G1/G2), 56 and 6 (for G1/G3) and 53 and 6 (for G2/G3) features. Refer to Supplementary Tables S5–S7 for features remaining after step II and to Table 2 for the top five features selected after the MI-based feature selection step (step III).

### 3.2. Multivariable Prediction Models

**Figure 5:**
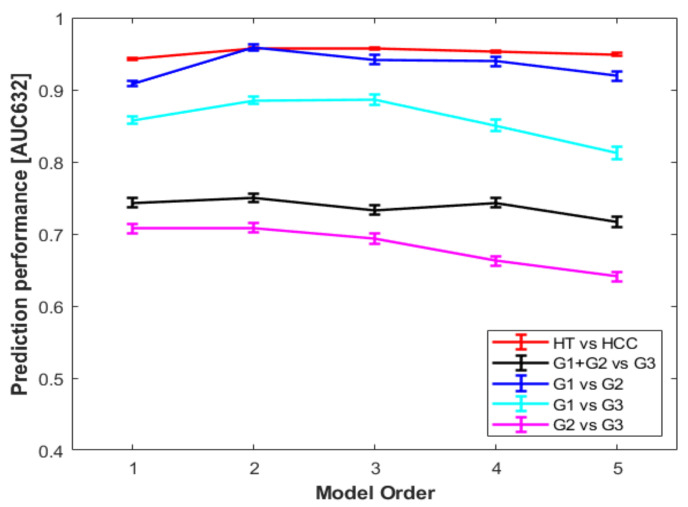
Prediction performances in terms of 0.632+ AUC of models from order 1 to 5 for each classification task. Abbreviations: HCC, hepatocellular carcinoma; HT, normal liver parenchyma; G1, well-differentiated HCC; G2, moderately differentiated HCC; G3, poorly differentiated HCC; AUC, area under the receiver operating characteristic curve.

Multivariable logistic regression models for the HCC/HT classification task revealed high prediction performances for any model order. However, based on Figure 5 and prediction performance metrics (Supplementary Table S8), the second order model was the simplest multivariable model with the best prediction performances was (AUC = 96%, sen = 94%, spec = 91%, and acc = 92% respectively). However, the DeLong test performed for each pair of models built for HCC/HT classification task was not significant (see Supplementary Table S13).

Concerning classification tasks for HCC grading, prediction performances were overall lower than those shown for the HCC/HT classification task. Based on Figure 5 and prediction performance metrics (Supplementary Table S9), we determined that the fourth-order model was the simplest multivariable one with the best prediction performances for G1 + G2/G3 classification task (AUC = 74%, sen = 64%, spec = 69%, acc = 67%, respectively) with respect to the first-order model and the higher-order models. For G1/G2 classification task, performances were overall higher across the five model orders in terms of AUC (90–95%), sensitivity (83–91%) and accuracy (79–88%). However, the model of order four was chosen due to a higher specificity (81%). Similar results were also obtained for models built for G1/G3 classification task, the inspection of which resulted in choosing the third-order model, due to higher performances compared with the other models (AUC = 89%, sen = 84%, spec = 76%, acc = 83%). Finally, the second-order model was chosen for G2/G3 classification task, although values of AUC, sensitivity specificity and accuracy were overall lower than those obtained for the other classification tasks (71%, 61%, 66%, 63%, respectively). Here again, the DeLong test performed for each pair of models built for grading classification tasks was not significant (see Supplementary Table S13). Prediction performances for each classification task are reported in Supplementary Tables S8–S12 and showed in Figure 5.

The computation of the multivariable model coefficients according to Equations (1) and (2) led to the following prediction models for HCC/HT, G1 + G2/G3, G1/G2, G1/G3 and G2/G3, classification tasks:

g_HCC/HT_(x_i_) = −21.4 × (T2 gldm Dependence Non Uniformity Normalized) + 7.41 × (T2 glrlm Long Run High Gray Level Emphasis) + 0.18, (3)

g_G1+G2/G3_(x_i_) = 0.62 × (PORT gldm Large Dependence Low Gray Level Emphasis) − 1.74 × (PORT glcm Maximum Probability) − 7.17 × (T2 glszm Low Gray Level Zone Emphasis) + 0.99 × (ART glszm Size Zone Non Uniformity Normalized) − 2.5, (4)

g_G1/G2_(x_i_) = −10.39 × (PORT ngtdm Strength) − 11.34 × (T2 gldm Low Gray Level Emphasis) − 8.98 × (ART firstorder 10Percentile) + 4.58 × (TARD firstorder Maximum) + 9.38, (5)

g_G1/G3_(x_i_) = 4.08 × (T2 gldm Large Dependence High Gray Level Emphasis) + 9.61 × (T2 gldm Large Dependence High Gray Level Emphasis) − 7.52 × (PORT glcm Maximum Probability) + 3.93, (6)

g_G2/G3_(x_i_) = 1.53 × (PORT ngtdm Complexity) − 1.59 × (PORT glszm Large Area Low Gray Level Emphasis) − 0.31 (7)

## 4. Discussion

In this study, we described a radiomics approach using preoperative T2 and arterial, portal, and tardive post-contrast images from DCE-MRI for detection and grading of HCC. Predictive radiomics signatures were separately built for five classification tasks, the first of which was designed to distinguish HCC from normal liver, and the remaining four to predict the aggressiveness of HCC based on the histopathological findings. Specifically, we assessed the predictive ability of radiomic features to distinguishing between well-, moderately and poorly differentiated HCC per pair. Moreover, we evaluated if radiomic features could be able to differentiate the combination of well- and moderately differentiated HCC from poorly differentiated HCC. Promising results were obtained from all five classification tasks, with best AUCs ranging from 71% to 96%. Prediction model for HCC/HT classification task showed high performances, with most relevant features arising from T2 and arterial phase of DCE-MRI, and almost all from the textural feature group. This could be related to the typical HCC dynamic enhancement pattern and hyperintensity on T2-weighted with respect to surrounding tissues and could be reflected by textural differences between HCC and normal liver parenchyma. Two features associated with GLRLM and GLDM matrices constituted the radiomic model for HCC/HT classification task. The GLRLM gave the size of homogeneous runs for each grey level and depicted intensity homogeneity in a given direction. The result might suggest that the intensity homogeneity between HT and HCC was different. Moreover, GLDM dependence non-uniformity normalized is associated with the homogeneity within the VOI and suggest that HCC and HT are different in terms of tissue homogeneity [53]. These results could be related to the discrepant microscopic features of HCC and HT. In particular, HT was more inclined to be uniform, while HCC was nonuniform due to cytological atypia and heterogeneity of cancerous cells. Although the literature is lacking in MRI radiomics studies aiming at distinguish normal hepatic tissue from HCC, textural features from both T2 and arterial phase of DCE-MRI were found to be able to characterize HCC from benign liver lesions, as well as other liver cancer types [62,63,64]. Starmans et al. [64] found that T2 radiomic features were able to predict liver tumor benignity with an AUC ranging from 0.75 to 0.92. T2 texture features were also found to be superior to qualitative diagnosis using DCE-MRI and DWI for distinguishing HCC from dysplastic nodules in study by Zhong et al. [60]. The 2D texture analysis performed by Stocker et al. [61] revealed that features from arterial phase were the most promising for distinguish HCC from benign lesions.

Only Hectors et al. investigated the power of histogram characteristics arising from multiparametric MRI in HCC and liver parenchyma. However, no textural features were investigated [65]. Moreover, Raman et al. built a textural-based radiomics model for distinguishing HCC and normal liver and found a 98.4% performance accuracy. However, the comparison with our results was not possible since this model was based on features extracted from CT. Moreover, while they evaluated normal tissue of healthy volunteers, in our study, HT regions were placed on healthy liver parenchyma of cancer patients [66].

Concerning the four classification tasks for prediction of HCC grading, performances were overall high for every selected model (AUC ranging from 71% to 95%), with features participating in model building mostly arising from second-order textural group. Higher performances were obtained from G1/G2 and G1/G3 classification tasks (best AUCs of 93% and 88%, respectively) with respect to those obtained from G1 + G2 vs. G3 and G2/G3 classification tasks, meaning that the models were better in distinguishing well-differentiated HCC from both moderately and poorly differentiated HCC than in distinguishing poorly differentiated HCC from both moderately differentiated and the well- and moderately differentiated HCC grouped together. Notably, textural features that contributed most to the prediction of HCC grade were GLCM, GLDM and GLSZM features. The GLCM is associated with pair-wise arrangement of pixels with the same gray-level and is then able to highlight local heterogeneity information. Therefore, it could be deduced that the different pathological grades might impact the gray value of the image. The GLDM and GLSZM, being associated with the homogeneity within the VOI and the size of homogeneous zone in the VOI, suggest that the intensity homogeneity between G1, G2 and G3 HCC was different [61]. On a physical basis, these results could be related to the discrepant microscopic features of G1, G2 and G3 HCC [4].

Features contributing to building models aimed at grading HCC involved not only features from arterial and T2, but also features from portal and tardive DCE-MRI phases reflecting radiological workflow where washin and washout provide valuable info to characterize and differentiate liver lesions.

These results were in line with those by Feng et al. who found that features from T2 and arterial phase were supposed to be important to predict the differentiated degree of HCC [37]. Different from our results, they did not find any relevant results relating to the association of features from portal phase with histological degree. In addition, Choi et al. found promising results from MRI texture analysis. However, different from us, they investigated textural features from T2 and only the arterial phase from DCE-MRI, but also those from apparent diffusion coefficient map [67]. Zhou et al. found that textural features from T2 and arterial phase of DCE-MRI were associated with the histological differentiation of HCC. However, they did not evaluate features arising from the portal and delayed DCE-MRI phases [38]. On the contrary, Hectors et al. found no significant association between DCE-MRI radiomics features and pathological grade [65,68]. A recent study by Yang et al. revealed that MRI-based radiomics signatures built using T1, T2 and postcontrast DCE-MR images were able to predict poorly differentiated HCC with an AUC ranging from 0.58 to 0.72 [69]. However, any information on selected features constituting the prediction models was provided, thus preventing further comparison with our results.

Although prediction models were mostly based on textural features, several first order histogram features were found to be associated with differentiation of HCC from normal liver, as well as its histopathological grading, and contributed to prediction models building. This was in line with consideration by Hectors et al. who found that histogram analysis of multiparametric MRI features was promising for non-invasive HCC characterization on the imaging, histologic and genomics levels [65]. In addition, Feng et al. found histogram-derived features arising from T2 and both arterial and portal phase from DCE-MRI. Notably, the 10th percentile of DCE-MRI arterial phase was found to be correlated with the differentiated HCC degree [37]. This feature was found to be relevant in the G1/G2 classification task also in our study.

Conversely, we found that shape features were the most inefficient since none of them contributed to building the most powerful predictive models in the explored classification tasks. This was in accordance with considerations made in previous radiomic studies, and it could be justified by changes in shape and volume depending on different stages during disease progression [37,70]. In contrast, the higher-order statistic features, specifically texture features, occupied a significant position and could provide more valuable information according to our results.

Although the HCC/HT classification task could be of lesser clinical impact than those related to HCC grading, the promising results obtained could help strengthen the power of second-order textural MRI features, which proved to be useful in different HCC management steps such as characterization, grading, prediction of survival, recurrence, and microvascular invasion [67,71,72,73].

To the best of our knowledge, this is the first radiomic study aiming at investigating the power of T2 and post-contrast images from DCE-MRI for both HCC detection and grading. To date, relative few studies have dealt with radiomic features extracted from MR images, mainly due to the difficulties in standardizing MRI acquisitions that are characterized by a huge number of acquisition parameters and variations across manufacturers [30].

Despite our encouraging results, our study suffers from several limitations. First, the patient population was too small and unbalanced to generalize results, mainly concerning the building of models for prediction of HCC grade. Only seven patients had well-differentiated HCC, and this has made the dataset used for G1/G2 and G1/G3 classification tasks unbalanced. A larger and more balanced study group is thus needed to better conduct a radiomic analysis and build more robust prediction models using part of the dataset for the training, and part for testing and validating the performance of the classifiers with external datasets [60,74,75]. However, the IABR strategy we used for model building and performance prediction is a common reliable approach in case of small and imbalanced datasets [57,59]. Another source of bias of this study was that information on contrast agent type, concentration and flow rate was not available for all patients and could affect lesion/background dynamic enhancement and signal [76,77]. Moreover, the existing lack of standardization in radiomic investigations, in terms of image acquisition, processes, segmentation methods, and radiomics analysis tools, could lead to discrepancies in radiomic feature measurements that are not due to underlying biological variations. Reproducibility of radiomic features is of key importance to clinical applications in the field of HCC. Given that different institutions use different imaging techniques and equipment, and that these differences can have a direct impact on radiomic features, efforts are needed to develop a consistent methodology for extracting and processing the features. Of note, we used Pyradiomics software [52] for feature extraction, which (i) is compliant with IBSI guidelines (which promote standardization of radiomic analysis [53,78]), (ii) allows for a reproducible extraction of radiomic features due to the parameter files that could be shared and re-used and (iii) can also be used starting from DICOM input images with the file name pointing to a DICOM Segmentation Image object, thus automatically obtaining radiomic features without any intermediate steps. This allows for a reproducible feature extraction that can be achieved under real clinical conditions that usually involve DICOM objects.

Moreover, detailed reporting and documentation of radiomics studies is essential in order to develop this emerging field in terms of clinical translation and to improve the reproducibility of study outcomes. The radiomics quality score (RQS) has been introduced to assess radiomics studies in terms of their compliance with best-practice procedures and to provide a reference guide for the drafting of manuscripts of radiomics studies [24]. Although we proceeded to report in detail all steps of radiomic workflow performed in our study, the RQS remained low, mainly due to the lack of a prospective design, the absence of a validation test, and the missing incorporation of features beyond radiomics (such as clinical and/or molecular data) within the models. This consideration is in line with results by Wakabayashi et al. who performed a quantitative review on radiomics in HCC and found that RQS of the investigated studies ranged from low to moderate, with a mean ± standard deviation of 8.35 ± 5.38 [36].

Notably, we used 3D VOIs for lesion segmentation, and this should reduce inter-reader variability by eliminating the need to select a single-slice corresponding to a portion of a lesion, as well as enable a comprehensive description of the lesion given the increased number of voxels considered for radiomic features computation [79]. However, manual segmentation of 3D ROIs is time- and labor-consuming and is prone to user variability. More accurate and automatic tumor segmentation tools are needed to improve the quality of the radiomic analysis in future works [24].

Finally, because of its higher sensitivity, better spatial resolution, and soft-tissue characterization, MRI may provide more robust texture features for tumor heterogeneity assessment than CT [80]. However, given that the image signal intensities of tissues are strongly influenced by the MR acquisition parameters and MR images are more prone to artifacts that affect the quantitative analysis of texture features; simulating the textural composition of tissues with MR images can be more complicated than with CT. As a result, MRI-based radiomics signatures may be more predictive of tumor heterogeneity than CT-based radiomics, but they may be more vulnerable to fluctuations in imaging parameters [35]. However, we normalized MRI raw images to account for the varying intensity ranges of MRI data and improve the robustness of radiomics features, as indicated by the IBSI guidelines [50,52,78,79].

Based on the obtained preliminary results, radiomics may be a suitable tool for personalized treatment of HCC patients. The non-invasive nature of this approach could complement or replace tumor biopsy and could also be used to develop new prognostic biomarkers useful for HCC detection and grading without the need for invasive procedures. However, it is difficult to translate radiomic results into clinical practice, mainly due to the missing standardization of radiomic workflow and the resulting heterogeneity among HCC radiomics studies. In the future, it will be important to perform analysis on a more consistent patient sample that will make it possible to validate models on a validation set and to test different machine learning models. Moreover, it will be important to establish reproducible and interpretable radiomic markers for diagnosis and grading of HCC and to combine radiomic data with clinical/laboratory information and other omics data such as genomic and pathomic data [81,82,83]. The integration between quantitative data at different scales (radiological, pathological, molecular) will surely improve diagnostics and molecular knowledge about HCC, and this would have direct implications in clinical decision-making process. Moreover, this could be useful for the validation of the radiomic approach in clinical practice as “virtual biopsy” and to discover genotype–phenotype correlations [84].

## 5. Conclusions

In conclusion, our preliminary results support the significant role of T2 and DCE-MRI radiomic features for HCC diagnosis and grading. This could provide additional information on the biological aggressiveness of HCC and could be of great clinical impact with a view to personalized options involving the most minimal invasive procedures. Further studies are required to investigate the generalizability of our models and translate our results into clinical practice. By demonstrating clinical utility and reproducibility, radiomics models can prove their potential as a clinical decision-making tool that facilitates HCC diagnosis and grading.

## Figures and Tables

**Table 1:** Characteristics of included patients. Abbreviations: SD, standard deviation; HBV, hepatitis B; HCV, hepatitis C; NAFLD, non-alcoholic fatty liver disease; BCP, birth control pills; RF, risk factors; PH, portal hypertension; Y, yes; N, no; G1, well-differentiated HCC; G2, moderately differentiated HCC; G3, poorly differentiated HCC; NA, not assessed; AJCC, American Joint Committee on Cancer.

Clinical Characteristic	Value
Age (mean ± SD)	57.8 ± 15.3
Sex (n (%))	
Male	26 (68.4)
Female	12 (31.6)
Risk factors (n (%))	
HBV	3 (7.9)
HBV|tobacco	2 (5.3)
HCV	5 (13.2)
HCV|tobacco	2 (5.3)
HCV|alcohol	1 (2.63)
Alcohol	9 (23.7)
Tobacco	1 (2.6)
Tobacco|BCP	1 (2.6)
NAFLD	2 (5.3)
Hemochromatosis	1 (2.6)
No history of RF	10 (26.3)
NA	1 (2.6)
PH ^1^ (n (%))	
Y	9 (23.7)
N	29 (76.3)
Histologic grade (n (%))	
G1	7 (18.4)
G2	15 (39.5)
G3	16 (42.1)
AJCC stage ^2^ (n (%))	
I	15 (39.5)
II	12 (31.6)
III	10 (26.3)
IV	1 (2.6)

^1^ A portal vein diameter greater than 13 mm was assumed to be the cutoff point for PH [44,45,46]. ^2^ The AJCC staging system (ranging from the 5th through the 7th edition) was applied to classify the pathologic staging [47,48,49].

**Table 2:** Top 5 selected features on the basis of the Mutual Information metric, for each classification task. In grey the features contributing to building the most powerful models. Abbreviations: HCC, hepatocellular carcinoma; HT, normal liver parenchyma; G1, well-differentiated HCC; G2, moderately differentiated HCC; G3, poorly differentiated HCC; T2, features extracted from T2 images; ART, features extracted from arterial post-contrast phase of DCE-MRI; PORT, features extracted from portal post-contrast phase of DCE-MRI; TARD, features extracted from tardive post-contrast phase of DCE-MRI.

Classification Task	Top 5 Selected Features
HCC/HT	T2 gldm Dependence Non Uniformity Normalized
	T2 glszm Small Area Low Gray Level Emphasis
	T2 glrlm Long Run High Gray Level Emphasis
	ART firstorder Minimum
	ART gldm Large Dependence Low Gray Level Emphasis
G1 + G2/G3	PORT gldm Large Dependence Low Gray Level Emphasis
	ART glszm Size Zone Non Uniformity Normalized
	PORT glcm Maximum Probability
	PORT glszm Small Area Low Gray Level Emphasis
	T2 glszm Low Gray Level Zone Emphasis
G1/G2	PORT ngtdm Strength
	T2 gldm Low Gray Level Emphasis
	ART firstorder 10Percentile
	ART firstorder Skewness
	TARD firstorder Maximum
G1/G3	SHAPE Surface Volume Ratio
	T2 gldm Large Dependence High Gray Level Emphasis
	PORT glcm Maximum Probability
	ART glcm Cluster Shade
	ART firstorder Skewness
G2/G3	PORT gldm Large Dependence Low Gray Level Emphasis
	PORT glszm Zone Percentage
	PORT ngtdm Complexity
	PORT glszm Large Area Low Gray Level Emphasis
	TARD glrlm Long Run Low Gray Level Emphasis

## Data Availability

The datasets for this study are available from The Cancer Imaging Archive (TCIA). Link: https://wiki.cancerimagingarchive.net/display/Public/TCGA-LIHC (accessed on 30 September 2021).

## Supplementary Materials

The following are available online at https://www.mdpi.com/article/10.3390/diagnostics12051085/s1, Section S1. Selected features after Spearman correlation filter and Wilcoxon rank-sum test steps of feature selection. Section S2. PyRadiomics parameter file. Section S3. Selected features after Spearman correlation filter and Wilcoxon rank-sum test steps of feature selection. Section S4. Prediction performances of multivariable logistic regression models. Section S5. Results of DeLong test with Bonferroni correction. Section S6. Supplementary analysis for AJCC stage prediction.
