# Supplementary material for: MRI-Based Radiomic Features Help Identify Lesions and Predict Histopathological Grade of Hepatocellular Carcinoma
Source: Diagnostics (Basel). 2022 Apr 26;12(5):1085. doi: 10.3390/diagnostics12051085 (PMC9139902; doi:10.3390/diagnostics12051085)
Supplement: Supplementary file 1 [file diagnostics-12-01085-s001.zip › diagnostics-1609787-supplementary.pdf]

Supplementary Materials

Section S1. Selected features after Spearman correlation filter and Wilcoxon rank-sum test steps of feature selection.

Feature Type	*Feature name*
**Shape**<br>**[n = 14]**	shape Flatness<br>shape Sphericity<br>Maximum2D Diameter Row<br>shape MeshVolume<br>shape Elongation<br>shape Maximum2DDiameterColumn<br>shape VoxelVolume<br>shape SurfaceArea<br>shape MinorAxisLength<br>shape Maximum3DDiameter<br>shape MajorAxisLength<br>shape LeastAxisLength<br>shape SurfaceVolumeRatio<br>shape Maximum2DDiameterSlice
**First Order**<br>**[n = 18]**	Skewness<br>Maximum<br>MeanAbsoluteDeviation<br>Kurtosis<br>Range<br>Energy<br>InterquartileRange<br>Variance<br>Mean<br>10Percentile<br>90Percentile<br>Uniformity<br>Median<br>RobustMeanAbsoluteDeviation<br>RootMeanSquared<br>Minimum<br>TotalEnergy<br>Entropy
**Second order (GLCM)**<br>**[n = 24]**	glcm SumSquares<br>glcm Contrast<br>glcm InverseVariance<br>glcm ClusterTendency<br>glcm DifferenceAverage<br>glcm Idm<br>glcm ClusterShade<br>glcm Imc2<br>glcm Idn<br>glcm ClusterProminence<br>glcm Id<br>glcm Imc1<br>glcm Autocorrelation<br>glcm SumEntropy<br>glcm MaximumProbability<br>glcm MCC<br>glcm Correlation<br>glcm JointEntropy

	glcm Idmn<br>glcm JointAverage<br>glcm SumAverage<br>glcm DifferenceEntropy<br>glcm DifferenceVariance<br>glcm JointEnergy
**Second Order (GLRLM)**<br>**[n = 16]**	glrlm ShortRunHighGrayLevelEmphasis<br>glrlm RunLengthNonUniformity<br>glrlm ShortRunEmphasis<br>glrlm LongRunLowGrayLevelEmphasis<br>glrlm RunPercentage<br>glrlm GrayLevelVariance<br>glrlm LowGrayLevelRunEmphasis<br>glrlm GrayLevelNonUniformity<br>glrlm RunVariance<br>glrlm LongRunHighGrayLevelEmphasis<br>glrlm ShortRunLowGrayLevelEmphasis<br>glrlm RunLengthNonUniformityNormalized<br>glrlm LongRunEmphasis<br>glrlm GrayLevelNonUniformityNormalized<br>glrlm HighGrayLevelRunEmphasis<br>glrlm RunEntropy
**Second Order (GLSZM)**<br>**[n = 16]**	glszm SizeZoneNonUniformity<br>glszm LargeAreaEmphasis<br>glszm SmallAreaEmphasis<br>glszm ZonePercentage<br>glszm GrayLevelVariance<br>glszm LargeAreaLowGrayLevelEmphasis<br>glszm GrayLevelNonUniformity<br>glszm SmallAreaHighGrayLevelEmphasis<br>glszm ZoneVariance<br>glszm LargeAreaHighGrayLevelEmphasis<br>glszm HighGrayLevelZoneEmphasis<br>glszm SizeZoneNonUniformityNormalized<br>glszm ZoneEntropy<br>glszm SmallAreaLowGrayLevelEmphasis<br>glszm GrayLevelNonUniformityNormalized<br>glszm LowGrayLevelZoneEmphasis
**Second Order (NGTDM)**<br>**[n = 5]**	ngtdm Coarseness<br>ngtdm Strength<br>ngtdm Busyness<br>ngtdm Complexity<br>ngtdm Contrast
**Second Order (GLDM)**<br><br>**[n = 14]**	gldm LargeDependenceLowGrayLevelEmphasis<br>gldm DependenceNonUniformity<br>gldm SmallDependenceLowGrayLevelEmphasis<br>gldm LowGrayLevelEmphasis<br>gldm LargeDependenceHighGrayLevelEmphasis<br>gldm DependenceEntropy<br>gldm DependenceVariance<br>gldm GrayLevelVariance<br>gldm GrayLevelNonUniformity<br>gldm SmallDependenceEmphasis<br>gldm LargeDependenceEmphasis

	gldm SmallDependenceHighGrayLevelEmphasis<br>gldm HighGrayLevelEmphasis<br>gldm DependenceNonUniformityNormalized

Table S1. Extracted radiomic features. First (n=18) and second order features (n = 75) were extracted from T2 and arterial, portal and tardive post-contrast phases of DCE-MRI images, for a total of 93 features for each of the four MRI images. For each VOI, a total of 386 radiomic features were extracted.

Section S2. PyRadiomics parameter file

```
#Brancato et al. - Parameter file for feature extraction with PyRadiomics - August 2021
# MRI-based radiomic features help identify lesions and predict histopathological grade of hepatocellular carcinoma

imageType:
  Original: {}

featureClass:
  shape:
  firstorder:
  glcm:
  glrlm:
  glszm:
  gldm:
  ngtdm:

setting:
  # Normalization:
  normalize: true
  normalizeScale: 100  # This allows you to use more or less the same bin width.

  # Image discretization:
  # The ideal number of bins is somewhere in the order of 16-128 bins. Binwidth was defined by extracting firstorder:Range from the dataset to analyze, and choosing a binwidth so, that range/binwidth remains approximately
  # in this range of bins (applied on T2, ART, PORT, TARD images).
  binWidth = 6

  # first order specific settings:
  # When normalizing, gray values below the mean will be negative. Shifting by 300 (3 StdDevs * 100) ensures that the
  # majority of voxels is positive (only outliers >3 SD lower than the mean will be negative).
  voxelArrayShift: 300

  # Misc:
  # label value
  label: 1
```

Section S3. Selected features after Spearman correlation filter and Wilcoxon rank-sum test steps of feature selection.

SHAPE shape Sphericity
SHAPE shape Elongation
ART glcm Cluster Shade
ART glrlm Long Run High Gray Level Emphasis
ART gldm Large Dependence Low Gray Level Emphasis
ART glszm Small Area Low Gray Level Emphasis
ART firstorder Minimum
ART firstorder Kurtosis
ART firstorder Skewness
PORT gldm Large Dependence Low Gray Level Emphasis
PORT glszm SmallAreaEmphasis
PORT glszm SmallAreaLowGrayLevelEmphasis
PORT firstorder Kurtosis
TARD glrlm RunVariance
TARD glszm SmallAreaEmphasis
TARD firstorder 10Percentile
TARD firstorder Kurtosis
T2 glcm Idn
T2 glcm MCC
T2 glcm Imc1
T2 glrlm ShortRunEmphasis
T2 glrlm LongRunHighGrayLevelEmphasis
T2 gldm DependenceNonUniformityNormalized
T2 glszm SizeZoneNonUniformityNormalized
T2 glszm SmallAreaLowGrayLevelEmphasis
T2 firstorder Minimum
ART ngtdm Strength
TARD ngtdm Strength
T2 ngtdm Strength

Table S2. 29 selected features after Wilcoxon rank-sum test for HCC/HT classification task.

SHAPE shape Sphericity
SHAPE shape Elongation
ART glcm ClusterShade
ART glrlm LongRunHighGrayLevelEmphasis
ART gldm LargeDependenceLowGrayLevelEmphasis
ART glszm SizeZoneNonUniformityNormalized
ART glszm SmallAreaLowGrayLevelEmphasis
ART firstorder Minimum
ART firstorder Kurtosis
ART firstorder Skewness
PORT gldm LargeDependenceLowGrayLevelEmphasis
PORT glszm SizeZoneNonUniformityNormalized
PORT glszm SmallAreaEmphasis
PORT glszm SmallAreaLowGrayLevelEmphasis

PORT firstorder Kurtosis
TARD glrlm RunVariance
TARD glszm SmallAreaEmphasis
TARD firstorder 10Percentile
TARD firstorder Kurtosis
T2 glcm Idn
T2 glcm MCC
T2 glcm Imc1
T2 glrlm ShortRunEmphasis
T2 glrlm LongRunHighGrayLevelEmphasis
T2 gldm LargeDependenceLowGrayLevelEmphasis
T2 gldm DependenceNonUniformityNormalized
T2 glszm SizeZoneNonUniformityNormalized
T2 glszm SmallAreaEmphasis
T2 glszm SmallAreaLowGrayLevelEmphasis
T2 firstorder 10Percentile
T2 firstorder Minimum
ART ngtdm Strength
TARD ngtdm Strength
T2 ngtdm Strength

Table S3. 34 selected features after paired Wilcoxon signed-rank test for HCC/HT classification task.

ART glszm SizeZoneNonUniformityNormalized
PORT glcm MaximumProbability
PORT gldm LargeDependenceLowGrayLevelEmphasis
PORT glszm SmallAreaLowGrayLevelEmphasis
TARD glszm SizeZoneNonUniformityNormalized
T2 glszm LowGrayLevelZoneEmphasis

Table S4. 6 selected features after Wilcoxon rank-sum test for G1+G2/G3 classification task.

ART firstorder 10Percentile
ART firstorder Skewness
PORT firstorder Skewness
TARD firstorder Maximum
T2 gldm LowGrayLevelEmphasis
PORT ngtdm Strength

Table S5. 6 selected features after Wilcoxon rank-sum test for G1/G2 classification task.

SHAPE shape SurfaceVolumeRatio
ART glcm ClusterShade
ART firstorder Skewness
PORT glcm MaximumProbability
TARD gldm LargeDependenceLowGrayLevelEmphasis
T2 gldm LargeDependenceHighGrayLevelEmphasis

Table S6. 6 selected features after Wilcoxon rank-sum test for G1/G3 classification task.

PORT glcm MaximumProbability
PORT gldm LargeDependenceLowGrayLevelEmphasis
PORT glszm ZonePercentage
PORT glszm LargeAreaLowGrayLevelEmphasis

TARD glrlm LongRunLowGrayLevelEmphasis	
PORT ngtdm Complexity	

Table S7. 6 selected features after Wilcoxon rank-sum test for G2/G3 classification task.

Section S4. Prediction performances of multivariable logistic regression models.

Order	Features	AUC ± SE	SEN ± SE	SPEC ± SE	ACC ± SE	MCC ± SE
1	T2 gldm DNUN	0.943 ± 0.002	0.895 ± 0.004	0.9 ± 0.003	0.897 ± 0.002	0.695 ± 0.005
2	T2 gldm DNUN<br>T2 glrlm LRHGLE	0.958 ± 0.001	0.939 ± 0.003	0.905 ± 0.002	0.919 ± 0.002	0.768 ± 0.004
3	T2 gldm DNUN<br>T2 glrlm LRHGLE<br>ART firstorder Minimum	0.957 ± 0.001	0.926 ± 0.004	0.903 ± 0.002	0.913 ± 0.002	0.731 ± 0.005
4	T2 gldm DNUN<br>T2 glrlm LRHGLE<br>ART firstorder Minimum<br>ART gldm LDLGLE	0.953 ± 0.002	0.907 ± 0.005	0.917 ± 0.003	0.914 ± 0.002	0.733 ± 0.005
5	T2 gldm DNUN<br>T2 glrlm LRHGLE<br>ART firstorder Minimum<br>ART gldm LDLGLE<br>T2 glszm SALGLE	0.949 ± 0.002	0.899 ± 0.005	0.903 ± 0.003	0.903 ± 0.002	0.739 ± 0.006

Table S8. Results of multivariate analysis for HT/HCC classification task. For each model (from order 1 to 5), AUC, sensitivity, specificity and accuracy were reported with the standard error on a 95% confidence interval over all bootstrap sample. Abbreviations: T2 = features extracted from T2 images; ART = features extracted from arterial post-contrast phase of DCE-MRI; PORT = features extracted from portal post-contrast phase of DCE-MRI; TARD = features extracted from tardive post-contrast phase of DCE-MRI; DNNU = Dependence Non Uniformity Normalized; LRHGLE = Long Run High Gray Level Emphasis; LDLGLE = Large Dependence Low Gray Level Emphasis; SALGLE = Small Area Low Gray Level Emphasis.

Order	Features	AUC ± SE	SEN ± SE	SPEC ± SE	ACC ± SE	MCC ± SE
1	PORT glcm MP	0.743 ± 0.006	0.668 ± 0.015	0.564 ± 0.013	0.605 ± 0.006	0.224 ± 0.02
2	ART glszm SZNUN<br>T2 glszm LGLZE	0.75 ± 0.006	0.644 ± 0.011	0.647 ± 0.009	0.643 ± 0.005	0.263 ± 0.017
3	ART glszm SZNUN<br>T2 glszm LGLZE<br>PORT glcm MP	0.733 ± 0.006	0.638 ± 0.011	0.677 ± 0.009	0.66 ± 0.005	0.3 ± 0.016
4	PORT gldm LDLGLE<br>PORT glcm MP<br>T2 glszm LGLZE<br>ART glszm SZNUN	0.743 ± 0.007	0.638 ± 0.012	0.688 ± 0.009	0.668 ± 0.005	0.278 ± 0.02
5	PORT gldm LDLGLE<br>PORT glcm MP<br>T2 glszm LGLZE<br>ART glszm SZNUN<br>PORT glszm SALGLE	0.717 ± 0.007	0.633 ± 0.012	0.69 ± 0.009	0.669 ± 0.005	0.234 ± 0.047

Table S9. Results of multivariate analysis for G1+G2/G3 classification task. For each model (from order 1 to 5), AUC, sensitivity, specificity and accuracy were reported with the standard error on a 95% confidence interval over all

bootstrap sample. Abbreviations: T2 = features extracted from T2 images; ART = features extracted from arterial post-contrast phase of DCE-MRI; PORT = features extracted from portal post-contrast phase of DCE-MRI; TARD = features extracted from tardive post-contrast phase of DCE-MRI; LDLGLE = Large Dependence Low Gray Level Emphasis; MP = Maximum Probability; LGLZE = Low Gray Level Zone Emphasis; SZNUN = Size Zone Non Uniformity Normalized; SALGLE = Small Area Low Gray Level Emphasis.

Order	Features	AUC ± SE	SEN ± SE	SPEC ± SE	ACC ± SE	MCC ± SE
1	PORT ngtdm Strength	0.909 ± 0.004	0.83 ± 0.006	0.665 ± 0.015	0.79 ± 0.005	0.468 ± 0.018
2	T2 gldm LGLE<br>ART firstorder 10Percentile	0.959 ± 0.004	0.894 ± 0.007	0.736 ± 0.018	0.862 ± 0.005	0.631 ± 0.018
3	PORT ngtdm Strength<br>T2 gldm LGLE<br>ART firstorder 10Percentile	0.942 ± 0.006	0.916 ± 0.005	0.717 ± 0.022	0.879 ± 0.005	0.617 ± 0.023
4	PORT ngtdm Strength<br>T2 gldm LGLE<br>ART firstorder 10Percentile<br>TARD firstorder Maximum	0.939 ± 0.006	0.9 ± 0.007	0.807 ± 0.018	0.884 ± 0.006	0.664 ± 0.021
5	PORT ngtdm Strength<br>T2 gldm LGLE<br>ART firstorder 10Percentile<br>TARD firstorder Maximum<br>ART firstorder Skewness	0.919 ± 0.007	0.88 ± 0.007	0.798 ± 0.018	0.865 ± 0.006	0.609 ± 0.021

Table S10. Results of multivariate analysis for G1/G2 classification task. For each model (from order 1 to 5), AUC, sensitivity, specificity and accuracy were reported with the standard error on a 95% confidence interval over all bootstrap sample. Abbreviations: T2 = features extracted from T2 images; ART = features extracted from arterial post-contrast phase of DCE-MRI; PORT = features extracted from portal post-contrast phase of DCE-MRI; TARD = features extracted from tardive post-contrast phase of DCE-MRI; LGLE = Low Gray Level Emphasis.

Order	Features	AUC ± SE	SEN ± SE	SPEC ± SE	ACC ± SE	MCC ± SE
1	ART firstorder Skewness	0.858 ± 0.005	0.826 ± 0.007	0.508 ± 0.016	0.747 ± 0.005	0.264 ± 0.05
2	PORT glcm MP<br>ART firstorder Skewness	0.885 ± 0.005	0.826 ± 0.006	0.7 ± 0.016	0.799 ± 0.004	0.489 ± 0.016
3	T2 gldm LDHGLE<br>ART firstorder Skewness<br>PORT glcm MP	0.886 ± 0.007	0.843 ± 0.009	0.755 ± 0.015	0.828 ± 0.006	0.583 ± 0.016
4	T2 gldm LDHGLE<br>ART firstorder Skewness<br>PORT glcm MP<br>ART glcm CS	0.851 ± 0.008	0.783 ± 0.01	0.745 ± 0.016	0.782 ± 0.007	0.484 ± 0.02
5	ART firstorder Skewness<br>PORT glcm MP<br>T2 gldm LDHGLE<br>ART glcm CS<br>SHAPE shape SVR	0.812 ± 0.009	0.741 ± 0.011	0.702 ± 0.017	0.742 ± 0.008	0.354 ± 0.025

Table S11. Results of multivariate analysis for G1 /G3 classification task. For each model (from order 1 to 5), AUC, sensitivity, specificity and accuracy were reported with the standard error on a 95% confidence interval over all bootstrap sample. Abbreviations: T2 = features extracted from T2 images; ART = features extracted from arterial post-

contrast phase of DCE-MRI; PORT = features extracted from portal post-contrast phase of DCE-MRI; MP = Maximum Probability; SVR = Surface Volume Ratio; LDHGLE = Large Dependence High Gray Level Emphasis; CS = Cluster Shade.

Order	Features	AUC ± SE	SEN ± SE	SPEC ± SE	ACC ± SE	MCC ± SE
1	PORT ngtdm Complexity	0.707 ± 0.006	0.569 ± 0.01	0.661 ± 0.012	0.609 ± 0.005	0.165 ± 0.06
2	PORT ngtdm Complexity<br>PORT glszm LALGLE	0.709 ± 0.006	0.609 ± 0.01	0.664 ± 0.013	0.63 ± 0.006	0.237 ± 0.026
3	PORT ngtdm Complexity<br>PORT glszm LALGLE<br>TARD glrlm LRLGLE	0.693 ± 0.007	0.605 ± 0.009	0.674 ± 0.012	0.638 ± 0.005	0.215 ± 0.029
4	PORT ngtdm Complexity<br>PORT glszm LALGLE<br>TARD glrlm LRLGLE<br>PORT gldm LDLGLE	0.663 ± 0.006	0.613 ± 0.009	0.639 ± 0.013	0.628 ± 0.006	0.187 ± 0.03
5	PORT gldm LDLGLE<br>PORT ngtdm Complexity<br>PORT glszm ZP<br>TARD glrlm LRLGLE<br>PORT glszm LALGLE	0.641 ± 0.007	0.616 ± 0.01	0.598 ± 0.013	0.614 ± 0.006	0.103 ± 0.033

Table S12. Results of multivariate analysis for G2/G3 classification task. For each model (from order 1 to 5), AUC, sensitivity, specificity and accuracy were reported with the standard error on a 95% confidence interval over all bootstrap sample. Abbreviations: T2 = features extracted from T2 images; ART = features extracted from arterial post-contrast phase of DCE-MRI; PORT = features extracted from portal post-contrast phase of DCE-MRI; LALGLE = Large Area Low Gray Level Emphasis; LRLGLE = Long Run Low Gray Level Emphasis; ZP = Zone Percentage; LDLGLE = Large Dependence Low Gray Level Emphasis.

Section S5. Results of DeLong test with Bonferroni correction.

**TASK – HCC vs HT**		
Models	p	95% CI
1-2	0.303	0-0.12
1-3	0.393	0-0.114
1-4	0.282	0-0.114
1-5	0.244	0-0.032
2-3	0.34	0-0.034
2-4	0.372	0-0.017
2-5	0.346	0-0.18
3-4	0.4	0-0.058
3-5	0.271	0-0.141
4-5	0.362	0-0.033
**TASK – G1+G2 vs G3**		
Models	p	95% CI
1-2	0.762	0-0.16
1-3	0.387	0-0.128
1-4	0.234	0-0.111
1-5	0.211	0-0.116
2-3	0.196	0-0.064

2-4	0.293	0-0.086
2-5	0.315	0-0.097
3-4	0.645	0-0.045
3-5	0.599	0-0.066
4-5	0.726	0-0.04
**TASK – G1 vs G2**		
Models	p	95% CI
1-2	0.271	0-0.141
1-3	0.199	0-0.141
1-4	0.152	0-0.144
1-5	0.152	0-0.144
2-3	0.4	0-0.031
2-4	0.332	0-0.053
2-5	0.332	0-0.053
3-4	0.4	0-0.031
3-5	0.4	0-0.031
4-5	0.4	0-0.031
**TASK – G1 vs G3**		
Models	p	95% CI
1-2	0.34	0-0.178
1-3	0.299	0-0.187
1-4	0.552	0-0.204
1-5	0.346	0-0.18
2-3	0.4	0-0.058
2-4	0.742	0-0.147
2-5	0.828	0-0.112
3-4	0.607	0-0.141
3-5	0.816	0-0.104
4-5	0.105	0-0.315
**TASK – G2 vs G3**		
Models	p	95% CI
1-2	0.34	0-0.178
1-3	0.299	0-0.187
1-4	0.552	0-0.204
1-5	0.346	0-0.18
2-3	0.4	0-0.058
2-4	0.742	0-0.147
2-5	0.828	0-0.112
3-4	0.607	0-0.141
3-5	0.816	0-0.104
4-5	0.105	0-0.315

Table S13. Results of DeLong test with Bonferroni correction for each pair of models built for each classification task. p-values (p) for the difference in AUC and 95 % confidence interval (CI) for the difference in AUCs were reported.

Section S6. Supplementary analysis for AJCC stage prediction.

In order to investigate the ability of T2 and DCE-MRI radiomic features in predicting AJCC stage, two classification tasks were investigated: stage I and II versus stage III and IV (I-II/III-IV classification task) and and stage I versus II, III, and IV (I/II-III-IV classification task). Procedures described in the "Radiomic feature selection" and "Multivariable prediction model building and analysis" paragraphs were used. As reported in the following Tables, the step I and step II of feature selection returned respectively 51 and 10 (for I-II/III-IV), 53 and 8 (for I/II-III-IV). For I-II/III-IV classification task, the simplest multivariable model best prediction performances were reached by the second order model (AUC = 86%, sen = 65%, spec = 82%, acc = 77%). For I-II/III-IV classification task, the simplest multivariable model best prediction performances were reached by the second order model (AUC = 77%, sen = 75%, spec = 65%, acc = 72%). However, these

results must be interpreted with caution since there were multiple changes to the AJCC classifcation over the time period during which HCC specimens were collected (resulting in differences in the pathologic AJCC staging criteria across different editions). This constitutes a limitation in using AJCC stage as outcome. In particular, the AJCC TNM classifcations used across cases in the TCGA-LIHC dataset range from the 5th through the 7th editions, resulting in inconsistency in the meaning of the pathologic T, N, and M categories across different patients examined during different time periods. These inconsistencies could also justify the differences in prediction performances obtained when the early stage group included stage I and II patients with respect to those obtained when the early stage group included only stage I patients.

S6.2 Prediction performances of multivariable logistic regression models.

ART_original_glcm_InverseVariance
ART_original_firstorder_Skewness
PORT_original_firstorder_10Percentile
PORT_original_firstorder_Kurtosis
PORT_original_firstorder_Skewness
TARD_original_firstorder_Kurtosis
T2_original_glcm_InverseVariance
T2_original_gldm_DependenceNonUniformityNormalized
T2_original_glszm_SizeZoneNonUniformityNormalized
T2_original_glszm_SmallAreaEmphasis

Table S14. 10 selected features after Wilcoxon rank-sum test for I-II/III-IV classification task.

SHAPE_original_shape_SurfaceVolumeRatio
ART_original_firstorder_Skewness
PORT_original_glszm_SizeZoneNonUniformityNormalized
PORT_original_firstorder_10Percentile
TARD_original_glszm_SizeZoneNonUniformityNormalized
TARD_original_firstorder_Skewness
T2_original_glszm_SizeZoneNonUniformityNormalized
T2_original_glszm_SmallAreaHighGrayLevelEmphasis

Table S15. 8 selected features after Wilcoxon rank-sum test for I/II-III-IV classification task.

S6.2 Prediction performances of multivariable logistic regression models.

Order	Features	AUC ± SE	SEN ± SE	SPEC ± SE	ACC ± SE	MCC ± SE
1	T2 glszm SZNUN	0.818 ± 0.004	0.517 ± 0.016	0.808 ± 0.006	0.744 ± 0.004	0.258 ± 0.019
2	T2 glszm SZNUN<br>PORT firstorder<br>10Percentile	0.859 ± 0.005	0.646 ± 0.013	0.815 ± 0.005	0.775 ± 0.004	0.386 ± 0.015
3	T2 glszm SZNUN<br>PORT firstorder<br>10Percentile<br>PORT firstorder<br>Kurtosis	0.857 ± 0.005	0.656 ± 0.014	0.795 ± 0.006	0.765 ± 0.004	0.381 ± 0.016
4	T2 glszm SZNUN<br>PORT firstorder<br>10Percentile<br>PORT firstorder<br>Kurtosis<br>T2 glszm SAE	0.82 ± 0.007	0.61 ± 0.015	0.789 ± 0.006	0.753 ± 0.005	0.328 ± 0.017
5	T2 glszm SZNUN<br>PORT firstorder	0.788 ± 0.008	0.6 ± 0.016	0.779 ± 0.006	0.743 ± 0.005	0.283 ± 0.018

	10Percentile<br>PORT firstorder Kurtosis<br>T2 glszm SAE<br>ART glcm IV					

Table S16. Results of multivariate analysis for I-II/III-IV classification task. For each model (from order 1 to 5), AUC, sensitivity, specificity and accuracy were reported with the standard error on a 95% confidence interval over all bootstrap sample. Abbreviations: T2 = features extracted from T2 images; ART = features extracted from arterial post-contrast phase of DCE-MRI; PORT = features extracted from portal post-contrast phase of DCE-MRI; SZNUN = Size Zone Non Uniformity Normalized; SAE = Small Area Emphasis; IV = Inverse Variance.

Order	Features	AUC ± SE	SEN ± SE	SPEC ± SE	ACC ± SE	MCC ± SE
1	PORT firstorder 10Percentile	0.737 ± 0.006	0.709 ± 0.01	0.561 ± 0.012	0.655 ± 0.006	0.265 ± 0.017
2	TARD firstorder Skewness<br>PORT firstorder 10Percentile	0.762 ± 0.006	0.746 ± 0.008	0.565 ± 0.012	0.683 ± 0.005	0.293 ± 0.016
3	T2 glszm SAHGLE<br>TARD firstorder Skewness<br>PORT firstorder 10Percentile	0.771 ± 0.007	0.752 ± 0.007	0.645 ± 0.012	0.715 ± 0.005	0.372 ± 0.016
4	T2 glszm SAHGLE<br>TARD firstorder Skewness<br>PORT firstorder 10Percentile<br>SHAPE shape SVR	0.751 ± 0.007	0.725 ± 0.007	0.622 ± 0.012	0.691 ± 0.005	0.29 ± 0.018
5	T2 glszm SAHGLE<br>TARD firstorder Skewness<br>PORT firstorder 10Percentile<br>SHAPE shape SVR<br>ART firstorder Skewness	0.723 ± 0.008	0.725 ± 0.007	0.603 ± 0.013	0.687 ± 0.005	0.264 ± 0.019

Table S17. Results of multivariate analysis for I/II-III-IV classification task. For each model (from order 1 to 5), AUC, sensitivity, specificity and accuracy were reported with the standard error on a 95% confidence interval over all bootstrap sample. Abbreviations: T2 = features extracted from T2 images; ART = features extracted from arterial post-contrast phase of DCE-MRI; PORT = features extracted from portal post-contrast phase of DCE-MRI; SAHGLE = Small Area High Gray Level Emphasis; SVR = Surface Volume Ratio.

S6.3 Equations for predictive logistic regression models.

$g_{I-II/III-IV}(x_i)$= 2.04×(PORT ngtdm Complexity)-1.77×(PORT glszm Large Area Low Gray Level Emphasis)-0.64

$g_{I/II-III-IV}(x_i)$= 1.24×(T2 glszm SAHGLE)+1.16×(TARD firstorder Skewness)-1.15×(PORT firstorder 10Percentile)-0.52

S6.4 Results of DeLong test with Bonferroni correction for each pair of models built for each classification task (I-II/III-IV and I/II-III-IV).

TASK – I+II vs III+IV		
Models	p	95% CI

1-2	0.122	0-0.101
1-3	0.118	0-0.110
1-4	0.203	0-0.105
1-5	0.145	0-0.113
2-3	0.773	0-0.0540
2-4	0.726	0-0.0450
2-5	0.884	0-0.0540
3-4	0.332	0-0.0320
3-5	0.779	0-0.0280
4-5	0.308	0-0.0380
**TASK – I vs II+III+IV**		
Models	p	95% CI
1-2	0.324	0-0.137
1-3	0.204	0-0.17
1-4	0.212	0-0.179
1-5	0.191	0-0.181
2-3	0.269	0-0.08
2-4	0.198	0-0.084
2-5	0.248	0-0.088
3-4	0.828	0-0.031
3-5	0.884	0-0.046
4-5	0.515	0-0.031
